# Observation of sonified movements engages a basal ganglia frontocortical network

**DOI:** 10.1186/1471-2202-14-32

**Published:** 2013-03-14

**Authors:** Gerd Schmitz, Bahram Mohammadi, Anke Hammer, Marcus Heldmann, Amir Samii, Thomas F Münte, Alfred O Effenberg

**Affiliations:** 1 Institute of Sports Science, University of Hannover, Hannover, Germany; 2 Department of Neurology, University of Lübeck, Lübeck, Germany; 3 International Neuroscience Institute, Hannover, Germany; 4 Department of Psychiatry, University of Erlangen, Erlangen, Germany

## Abstract

**Background:**

Producing sounds by a musical instrument can lead to audiomotor coupling, i.e. the joint activation of the auditory and motor system, even when only one modality is probed. The sonification of otherwise mute movements by sounds based on kinematic parameters of the movement has been shown to improve motor performance and perception of movements.

**Results:**

Here we demonstrate in a group of healthy young non-athletes that congruently (sounds match visual movement kinematics) vs. incongruently (no match) sonified breaststroke movements of a human avatar lead to better perceptual judgement of small differences in movement velocity. Moreover, functional magnetic resonance imaging revealed enhanced activity in superior and medial posterior temporal regions including the superior temporal sulcus, known as an important multisensory integration site, as well as the insula bilaterally and the precentral gyrus on the right side. Functional connectivity analysis revealed pronounced connectivity of the STS with the basal ganglia and thalamus as well as frontal motor regions for the congruent stimuli. This was not seen to the same extent for the incongruent stimuli.

**Conclusions:**

We conclude that sonification of movements amplifies the activity of the human action observation system including subcortical structures of the motor loop. Sonification may thus be an important method to enhance training and therapy effects in sports science and neurological rehabilitation.

## Background

In 1949, the famous Canadian neuroscientist Donald Hebb coined the phrase “Neurons that fire together wire together”, also known as Hebb’s axiom, implying that all aspects of an experience give rise to an amalgamated pattern of neural activity, which, if repeated, becomes entrained and more easily elicited.

A case in point of such integrated neural activity shaped by excessive and repeated experience has been auditory-motor coupling in the musician’s brain. Musicians create intricate sound-patterns by the movement of their hands. Sounds and movements are thus tightly coupled. Indeed, Haueisen and Knösche [1], using magnetoencephalography, showed that pianists who merely listened to pieces of well-trained piano music showed activation of the contralateral motor cortex. Similar observations have been made by a number of other researchers [2-7]. An important study by Bangert and co-workers compared professional pianists and non-musicians as they either listened to trained music or performed a short piece of music on a muted piano keyboard while lying in a scanner. The network recruited by professional musicians for listening to music as well as for performing musical actions was highly similar, suggesting transmodal co-activation. This network was speculated to have properties of a transmodal mirror neuron system [7]. Another example of coupling between motor and auditory brain areas has been reported by Lotze and co-workers [2] who compared fMRI activations of professional and amateur violinists during actual and imagined performance of a violin concerto. Besides activations in motor areas, professionals exhibited higher activity of the right primary auditory cortex during silent execution indicating increased audio-motor associative connectivity. Motor and auditory systems were coactivated in this study and co-activation was modulated as a function of musical training. To pinpoint the areas involved in audiomotor coupling Baumann et al. [5] investigated skilled pianists and non-musicians during silent piano performance and motionless listening to piano sound. A network of secondary and higher order auditory and motor areas was observed for both conditions among which the lateral dorsal premotor cortex and the pre-supplementary motor cortex (preSMA) played a significant role. While the majority of studies on audiomotor coupling has employed musical stimuli, Baumann and Greenlee [4] investigated real-life moving objects characterized by multisensory information. Random dot patterns moving in phase, moving out-of-phase, or being stationary were accompanied by auditory noise moving in phase, moving out-of-phase, or not moving. When the sound source was in phase with the visual coherent dot motion, performance of the participants was best. FMRI showed that auditory motion activated (among other regions) the superior temporal gyrus (STG) on the right more than on the left. Combined audiovisual motion activated the STG, the supramarginal gyrus, the superior parietal lobule, and the cerebellum.

One function of such integrated networks might be the facilitation of movement patterns. This notion has triggered interest, for example in the fields of sports science [8] or neurorehabilitation [9-11], to induce audiomotor coupling to enhance movement (re)-acquisition. The sonification of human movement patterns represents an approach to enrich movements - that are not normally associated with typical sound patterns - by adding an auditory component to the movement cycle [12,13]. This is achieved by transforming kinematic as well as dynamic movement parameters into sound. Emerging sound patterns are typical for a certain movement pattern. The additional movement acoustics can be exploited by multisensory integrative brain areas [8] and the transmodal mirror neuron system [7] which then might lead to a more stable and accurate representation of the movement. Congruent audiovisual motion information results in more accurate percepts, increased motor performance as well as enhanced motor learning. Behavioral benefits have been reviewed by Shams and Seitz [14,15] who argue that a larger set of processing structures is activated by multimodal stimuli. Moreover, Lahav et al. (2007) hypothesized an audiovisual mirror neuron system with premotor areas inherently involved and serving as an "action listening" and "hearing-doing mirror neuron system", with the latter being dependent on the individual's motor repertoire.

In learning new skills in sports or relearning basic skills in motor rehabilitation the observation of the skill and its reproduction are key elements. Observational motor learning can be achieved by visual perception, but vision is not the only sense providing information about movement patterns: especially in the temporal domain auditory perception is much more precise than visual perception. Unlike the movements of the pianist on the piano-keyboard, movements associated with running, swimming, or walking only give rise to little if any auditory information mostly limited to short movement phases, for example when the shoe hits the ground or the racket hits the ball. Even auxiliary auditory information provided by trainers or therapists is reduced to brief accents, such as clapping with the hands or the use of a drum. Previous research has indicated that continuous and more complex forms of auditory movement information like Audification or Sonification of naturally mute phases of movements can efficiently improve motor performance, e.g. when sonifying the inner hand pressure in freestyle swimming [16].

In the present study we first demonstrate that a movement sonification of breaststroke based on kinematic parameters leads to more precise judgements of swimming velocity differences when combined with a video of a breaststroke avatar. Second, to study the neural substrate of the effect of sonification on the perception of movements, fMRI activations to short video segments showing an avatar performing breaststroke movements accompanied either by congruent sounds, generated from kinematic parameters of the visual stimuli, or by incongruent sounds were studied in normal healthy volunteers. As in the behavioral experiment, participants had to compare two successive short video segments of a trial with regard to movement speed.

In addition to standard univariate analyses, fMRI was also analyzed using connectivity analysis [17]. We hypothesized that congruently sonified movements would engage additional brain areas relative to incongruent stimuli and that this network should, at least in part, coincide with brain areas identified as important for audiomotor integration.

## Methods

All procedures had been cleared by the ethics committee of the University of Magdeburg, the affiliation of the corresponding author at the time of the study.

### Participants

Seventeen student volunteers from different fields of study (7 women, age 24.6 years ± 4.4). At the time of testing none of the participants practiced swimming on a regular basis. Formerly, participants had engaged in regular swimming for 3.2 years (SD 4.1). Also, none of the participants could be considered expert musicians. Six of the participants never had learned to play an instrument. The mean number of years of active playing was 5.5 years (SD 6.1). All participants were healthy, right-handed native speakers of German with no history of neurological or psychiatric impairments. Basic visual and auditory abilities were normal as tested using a standard vision test for acuity and audiometry.

The subjects participated in a first behavioral session (I) and a second refreshing behavioral session (II) about five weeks later immediately prior to the fMRI session.

### Stimulus material

Behavioral as well as fMRI stimulus material was nearly identical, only differing in duration and inter-stimulus-interval.

**Figure 1:**
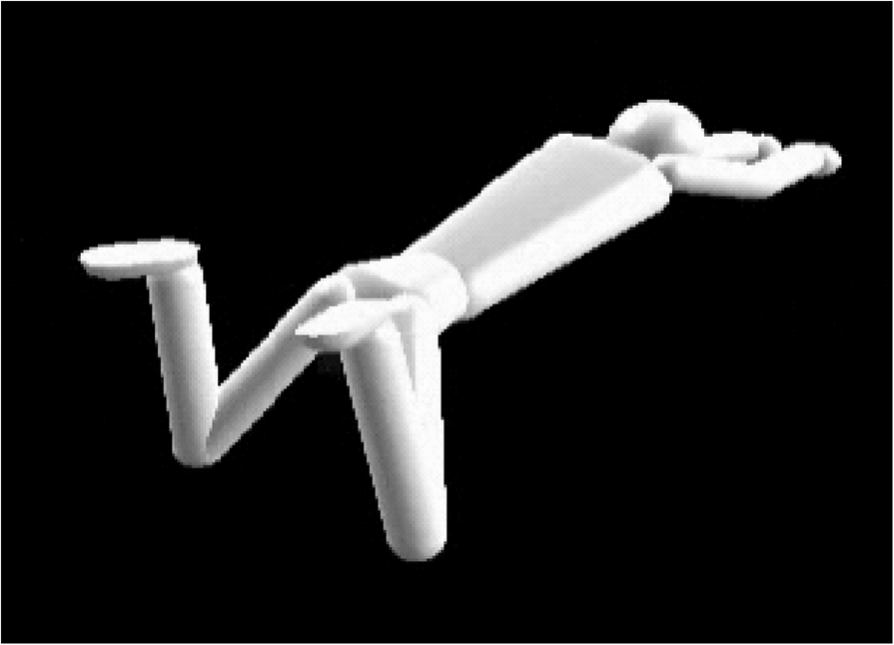
**Stimuli.** Visual stimulus component: a model of a solid swimmer performing breaststroke movements.

The visual stimulus component comprised a solid swimmer model performing breaststroke movements (Figure 1). Kinematics of the model were based on real human motion data and had been derived from 3D-video captures of a former breaststroke world champion. Absolute motion was eliminated by keeping the centre of the pelvis stationary. Therefore only relative motion was displayed. The congruent auditory stimulus component consisted of a movement sonification based on two kinematic parameters of the visual model: First, relative distance of the wrist joints to the centre of the pelvis was mapped to frequency of an electronic sound called "Fairlight Aahs". Moreover the relative velocity of this movement component was mapped to the loudness of the “Fairlight Aahs”. Both, velocity and loudness represent a joint intermodal elementary intensity category. The range of frequency modulation ("pitching") covered the interval between fis' and e''. Second, the relative distance of the ankle joints to the centre of the pelvis was mapped to the frequency of an electronic sound called "Pop Oohs". Again, the velocity of the movement was represented by the loudness of the sound. The pitch range covered the interval between contra B' and D. Both sounds were selected from the 'E-MU E4K' sound library. The kinematic-acoustic mapping was realized by using the 'Sonification-Tool'-Software [18] and provided a high degree of visual auditory stimulus convergence.

**Figure 2:**
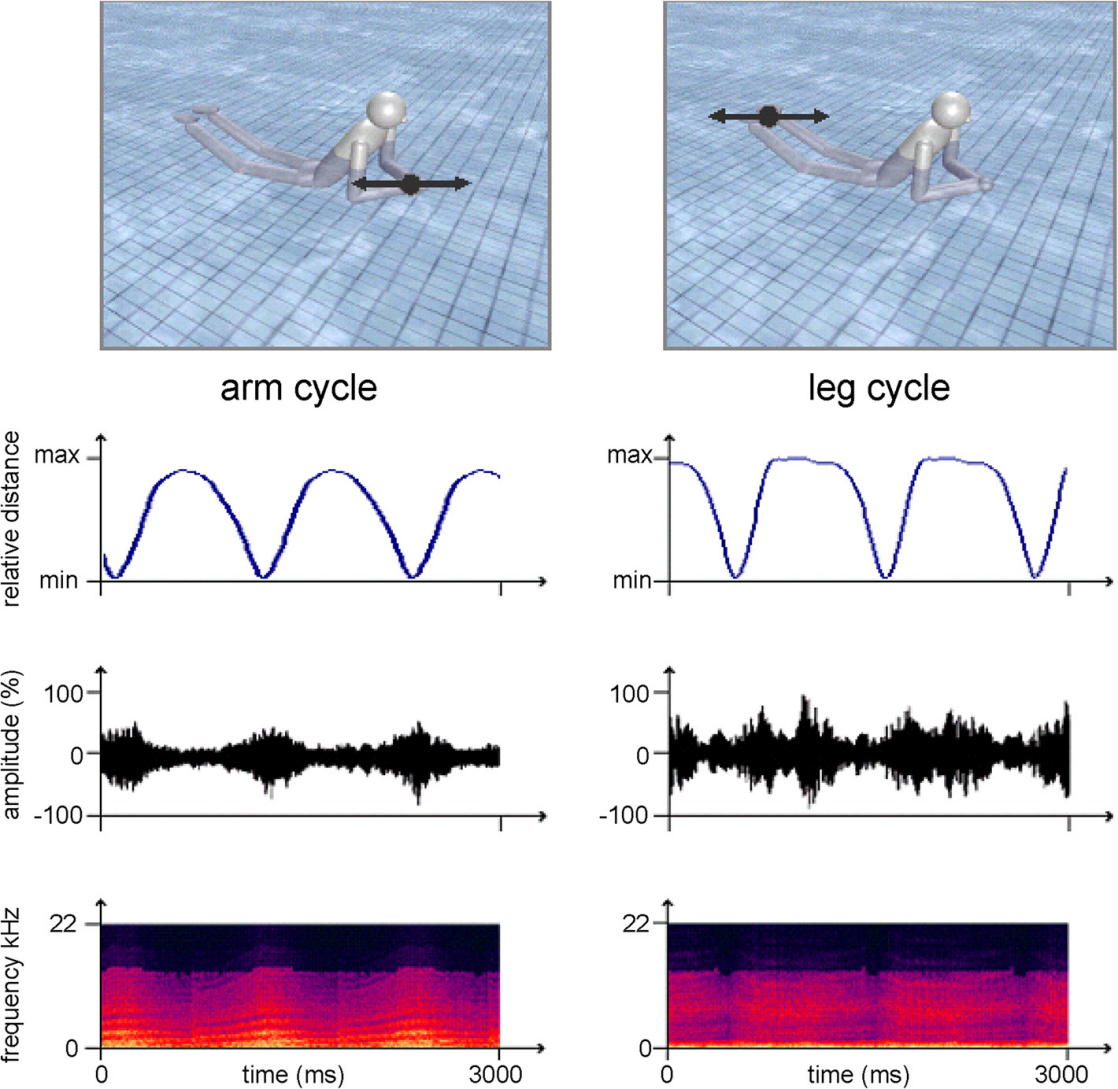
**Kinematic**-**acoustic mapping.** In the congruent condition frequency and amplitude modulations of electronic sounds represented changes in the relative distance between the wrist joints ("arm cycle", top and second row left) or the ankle joints ("leg-cycle", top and second row right) to the center of the pelvis. Third row: Sound pressure diagram; Fourth row: Spectrogram. Amplitude is color coded with cold / hot colors denoting low / high amplitudes.

Incongruent auditory information featured two different chords covering a similar timbre and pitch range as the congruent sonification over the course of a breaststroke. One chord lasted 1.0 s, 1.32 s, 1.8 s or 2.0 s and then changed into a second chord. As any kind of correspondence between chord switching and movement kinematics was avoided, the incongruent auditory information does not meet any criteria of a sonification. Details about the auditory part of the stimuli are given in Figure 2.

Original relative velocity of the audiovisual stimuli (100%) was varied in five steps (98%, 94%, 92%, 90% and 88%) to achieve subtle temporal variations of the swimming frequency. Those temporal variations were reduced to 98%, 94% and 92% in the fMRI session due to task requirements. The original kinematic data were interpolated and visualized with the 'Simba 2.0' Software to keep temporal continuity. Identical temporal variation was applied to the auditory stimuli: Sound sequences were stretched to 98%, 94%, 92%, 90% and 88% of the origin with 'cool edit 2.0' Software. Pitch frequency was preserved on stretching in order to enhance discrimination difficulty. To keep consistency of kinematic-acoustical mapping on the other hand – relative velocity of the swimmer model was mapped to sound amplitude and pitch frequency – pitch frequency was subsequently transposed marginally to 99%, 97%, 96%, 95% and 94% of the original.

### Procedure

A single trial consisted of two consecutive stimuli. Each stimulus contained of about five cycles of breast stroking in the behavioral session and was reduced to about two and a half cycles in the fMRI scanner session due to the temporal limitations of imaging studies. The duration of a single breast stroke cycle (at 100%) was 1.12 s. Absolute duration of a single stimulus was standardized to 6 s for the behavioral session and 3 s for the imaging session. The posture of the swim model at the first and the last picture of each stimulus was randomly varied to prevent an identification of a distinct stimulus based on initial and/or final posture. The inter-stimulus interval was set to 1.5 s (behavioral) or 0.5 s (imaging). The inter-trial interval lasted 6 s, providing 5 s for verbal response and 1 s for the indication to the next trial by presenting the trial number in the behavioural study. Inter-trial-interval was 11.5 s in the fMRI session allowing for the decline of the BOLD signal. In the fMRI study a manual response (pressing one of two buttons on an MRI congruent response pad) rather than a verbal response was used.

In behavioral session I the visual stimuli were projected on a 2.30 * 1.70 m sized screen located 4 m in front of the participants. In session II visual stimuli were displayed on a 0.37 * 0.23 m sized video-screen 0.5 m in front of the participants. Auditory stimuli were presented via headphones (beyerdynamic DT 100). Congruent and incongruent stimuli were arranged in blocks of 26 (session I) or 13 (session II) trials each. To investigate the perceptual effects of movement sonification, participants were instructed to estimate differences of swimming velocities between two consecutive breaststroke sequences. The mean absolute error (AE) of the absolute difference between the participants´ verbal response and the actual temporal difference of four breaststroke cycles from two consecutive sequences was chosen as dependent variable.

In the fMRI session visual stimuli were presented via MR-congruent video-goggles and the sound stimuli were presented by a shielded pneumatic headphone system with the sound level adapted such to be clearly audible against the scanner noise. The fMRI task required participants to judge whether the swimming velocities of stimulus 1 and 2 of a trial were “same” or “different” by pressing one of two buttons with the thumb of their right hand. A factorial design crossing the factors audiovisual congruency (congruent vs. incongruent) and velocity (same vs. different) was used. Twenty-four trials were presented for each of the 4 resulting conditions in random order.

### FMRI data acquisition and analysis

Data were collected on a 3-T Siemens Allegra system. Functional images were acquired using a T2*weighted echo planar imaging (EPI) sequence, with 2000-ms time repetition (TR), 30-ms time echo (TE), and 80° flip angle, in four runs. Each functional image consisted of 30 axial slices, with 64*64 matrix, 220 mm*220 mm field of view (FOV), 3.5-mm thickness, 0.35-mm gap, and 3.5 mm*3.5 mm in-plane resolution.

Structural images were acquired using a T1-weighted magnetization-prepared rapid-acquired gradient echo (MPRAGE) sequence, with 2500-ms TR, 1.68-ms TE, and 7° flip angle. The structural image consisted of 192 slices, with 256*256 matrix, 256 mm*256 mm FOV, 1-mm thickness, no gap, and 1 mm*1 mm in-plane resolution.

Data were analyzed with SPM8 (http://www.fil.ion.ucl.ac.uk/spm). The first four volumes were discarded owing to longitudinal magnetization equilibration effects. Functional images were first time-shifted with reference to the middle slice to correct differences in slice acquisition time. They were then realigned with a least squares approach and a rigid body spatial transformation to remove movement artifacts. Estimated movement parameters (six parameters per image: x, y, z, pitch, roll, and yaw) were included in GLMs as nuisance regressors of no interest to minimize signal-corrected motion effects. Realigned images were normalized to the EPI-derived MNI template (ICBM 152, Montreal Neurological Institute) and resampled to 2 mm × 2 mm × 2 mm voxel. Normalized images were smoothed with a Gaussian kernel of 8-mm full-width half-maximum (FWHM) and filtered with a high-pass filter of 128 s.

We carried out two statistical analyses, i.e. a standard univariate analysis and a functional connectivity analysis.

### Standard univariate analysis

The standard univariate analysis was performed to examine brain regions differentially activated in the processing of ‘*congruent*’ vs. ‘*incongruent*’ stimuli. Moreover, we also examined the effect of matching and non-matching stimulus pairs. This analysis was implemented on the basis of a GLM by using one covariate to model hemodynamic responses of all stimuli of a condition. Classical parameter estimation was applied with a one-lag autoregressive model to whiten temporal noise in fMRI time courses of each participant in order to reduce the number of false-positive voxels. The contrast maps were entered into two one-sample *t* tests on the group level. Resulting activation maps were considered at *p* < 0.05 (FDR-corrected) with a minimum cluster size of 10 voxels.

### Functional connectivity analysis

The functional connectivity analysis was performed to examine interregional interactions modulated in the processing of ‘*congruent*’ and ‘*incongruent*’ stimuli. This analysis was implemented on the basis of a GLM by using separate covariates to model hemodynamic responses of each single stimulus in each condition. Classical parameter estimation was applied with a one-lag autoregressive model. For each participant, estimated beta values were extracted to form a set of condition-specific beta series. The left STS (defined as a sphere of 5 mm around the activation peak in the univariate analysis) was defined as a seed region. Beta series of each seed were averaged across voxels within the critical region and correlated with beta series of every other voxel in the whole brain. Maps of correlation coefficients were calculated for each participant in each condition. The correlation maps were normalized with an arc-hyperbolic tangent transform and entered into two paired-sample *t* tests on the group level. Resulting connection maps were considered at *p* < 0.05 (FDR-corrected) with a minimum cluster size of 100 voxels. Two further seed regions were defined (right Brodmann area 6, right Brodmann area 44) but results will not be reported in this paper.

## Results

### Behavioral results

**Figure 3:**
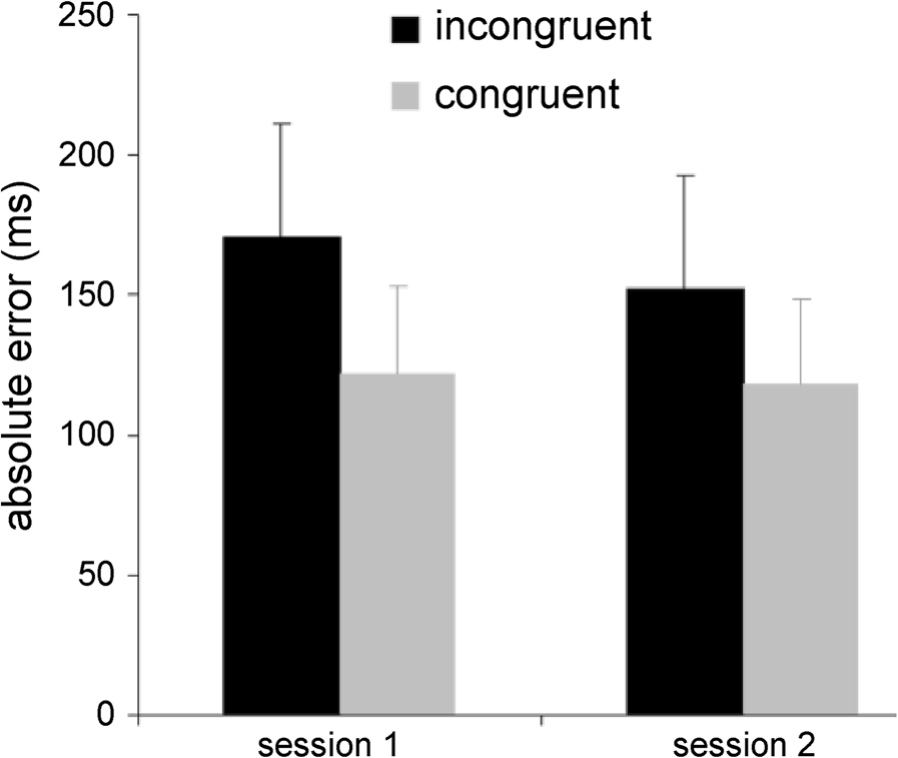
**Behavioral results.** Incongruent stimuli led to greater absolute error in both sessions. Error bars denote standard deviation.

The results of the two behavioral sessions are shown in Figure 3. AE was significantly lower in the congruent than the incongruent condition as confirmed by a two-way ANOVA with a significant effect condition (F_(1,16)_=25.93, p<0.001, η_p_^2^=0.62). Neither differences between both sessions nor the interaction were significant (session: F_(1,16)_=1.70, p>0.05, η_p_^2^=0.10; session*condition: F_(1,16)_=1.59, p>0.05, η_p_^2^=0.09). Therefore congruent audiovisual information led to more accurate perceptual judgements than incongruent audiovisual information.

### Imaging results

**Figure 4:**
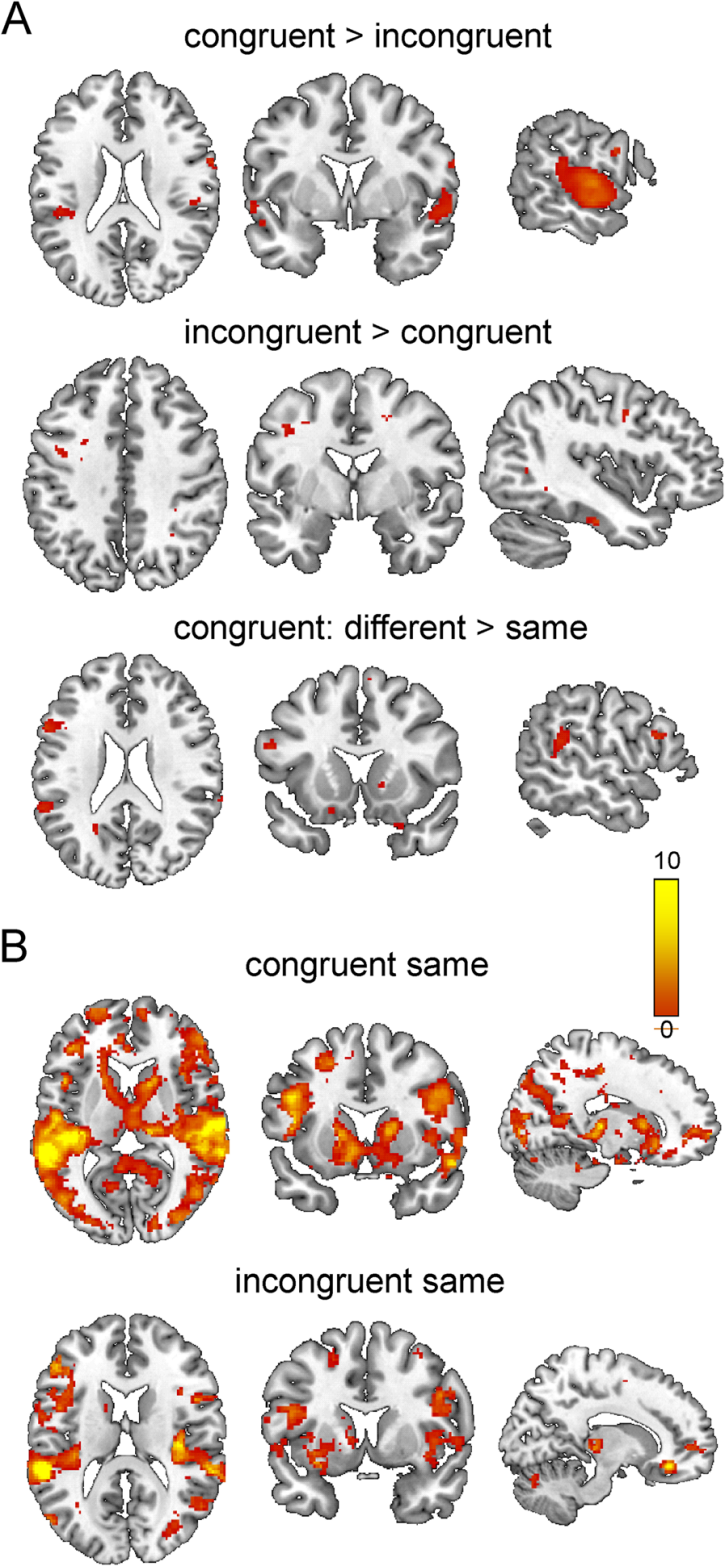
**fMRI results. A**: univariate analysis. FDR-corrected, p<0.05, minimum cluster size 10 voxels. **B**: Connectivity analysis using the left STS as a seed. For congruent stimuli more widespread connectivity is observed including frontal and parietal cortical areas as well as thalamus, caudate nucleus and putamen. FDR-corrected, p<0.05, minimum cluster size 100 voxels.

The results of the univariate analysis are shown in Figure 4A and Table 1. Congruent stimuli led to enhanced activity in superior and medial posterior temporal regions as well as the insula bilaterally and the precentral gyrus on the right side. Incongruent stimuli on the other hand were associated with more activity in the inferior temporal cortex (left), the frontal operculum (right), Brodmann area 6 (left) and the inferior parietal lobule. We also assessed activation differences between the congruent stimuli in which the two segments had different speeds vs. same speed. The former stimuli led to more activation in a number of brain areas as summarized in Table 1 and Figure 4A (bottom panel).

**Table 1:** Univariate analysis

**Region**	**BA**	**H**	**X**	**Y**	**Z**	***t***	**Size**
congruent > incongruent							
Sup & mid temporal, insula	21	L	−62	−8	−26	6.26	1955
Sup & mid temporal, insula	22	R	60	−10	2	6.81	2095
Precentral	6	R	62	4	24	5.06	23
Incongruent > congruent							
Inferior temporal	20	L	−42	−18	−26	5.25	17
Frontal operculum	44	R	44	6	22	4.20	29
	6	L	−42	0	40	4.43	20
Inf parietal lobule	40	R	48	42	46	4.49	19
congruent: different > same							
Superior temporal	21	R	66	−12	−8	4.99	24
Medial temporal	21	R	52	−32	−2	4.35	27
Inferior frontal gyrus	45	R	−44	30	2	4.34	30
Sup & mid temporal	48	L	−62	−42	28	4.86	198
Insula	48	R	34	−18	10	4.88	35
Inferior frontal gyrus	44	L	−56	18	20	5.05	54
Cingulate gyrus	-	L	−14	−22	36	6.50	134
Precentral	6	R	46	−12	56	4.70	54
Postcentral	4	L	−18	−36	72	4.66	24

*BA*, Brodmann Area; *H*, hemisphere; coordinates in MNI; *t*, statistic values; *L*, left; *R*, right; Size, number of voxels.

To assess the influence of sonification on network activity, connectivity analysis was performed using the left STS as a seed region separately for congruent and incongruent stimuli (same speed trials, Figure 4B, Tables 2 and 3). Clearly different connectivity patterns emerged for the congruent and incongruent stimuli. Whereas for congruent stimuli pronounced connectivity of the STS with the basal ganglia and thalamus as well as frontal regions was observed, this was not seen to the same extent for the incongruent stimuli.

**Table 2:** **Connectivity analysis**, **seed left STS**, **condition congruent** / **same**

**Cluster 1**	**xyz**	**Region**	**Size (voxel)**
T=22.18	−56 -38 12	left superior temporal cortex	37455
subcluster	sum classified voxels	subregion	
frontal	4909	precentral (r, 646), f~ inferior (r, 513), midf~ (r, 441), insula (r,440), medial orbitof~ (r, 358), gyrus rectus (r, 337; l, 257), inferior-f~ (l, 298), midf~ (l, 261), superior-f~ (l, 243), medial superior-f~ (r, 203), inferior orbitof~ (l, 189), medial orbitof~ (l, 184), superior f~ (r, 154), insula (l, 139), superior orbitof~ (l, 123; r, 123)	
temporal	2672	rolandic operculum (r, 455), inferior t~ (l, 375), t~ pole (r, 307), inferior t~ (r, 300), rolandic operculum (l, 202), hippocampus (r, 184), parahippocampus (r, 167; l, 139), angularis (r, 122), Heschl (r, 237; l, 184)	
occipital	1989	superior o~ (l, 631), calcarine sulcus (r, 319; l, 260), lingual (l, 249), inferior o~ (l, 204; r 192), lingual (r, 134)	
fusiform/cuneus	1578	fusiform (l, 502), cuneus (l, 429; r, 359), fusiform (r, 288),	
cingulate gyrus	1494	mid c~ (r, 584), anterior c~ (l, 516), posterior c~ (l, 228; r, 166)	
parietal	1445	superior p~ (l, 364; r, 324), inferior p~ (l, 303), supra marginalis (l, 204; r, 125), inferior p~ (r, 125)	
thalamus	695	thalamus (r, 482; l, 213)	
caudatus/putamen	684	caudatus (l, 438), putamen (l, 246),	
cerebellum	186	cerebellum (l, 186)	
**Cluster 2**	**x y z**	**region**	**size** (**voxel**)
T=10.18	−46 10 26	left frontal inferior cortex	2528
subcluster	sum classified voxels	subregion	
frontal	1714	mid f~ (l, 747), precentral (l, 400), inferior f~ (l, 355), superior f~ (l, 212)	
parietal	218	postcentral (l, 218)	
**Cluster 3**	**x y z**	**region**	**size** (**voxel**)
T=7.48	26 2 52	Left mid frontal cortex	236
**Cluster 4**	**x y z**	**region**	**size** (**voxel**)
T=6.72	8 -28 -28	Right cerebellum	107
subcluster	sum classified voxels	subregion	
Cerebellum/ brainstem	107	pons (90), cerebellum (l, 17)	

*T*, statistic values; *l*, left; *r*, right; size, number of voxels, *c*~ cingulate, *f*~ frontal, *t*~ temporal, *p*~ parietal, xyz coordinates of the peak voxel (MNI space).

**Table 3:** **Connectivity analysis**, **seed left STS**, **condition incongruent** / **same**

**Cluster 1**	**x y z**	**Region**	**size (voxel)**
T=21.88	−56 -38 12	left superior temporal cortex	4837
subcluster	sum classified voxels	subregion	
frontal		inferior f~ (l, 377), insula (l, 319), frontal inferior operculum (l, 269)	
temporal		superior t~(l, 1453), mid t~ (l, 663), rolandic operculum (l, 311)	
parietal		supra marginalis (l, 214), Heschl (l, 122)	
caudatus/putamen		putamen (l, 115)	
**Cluster 2**	**x y z**	**region**	**size** (**voxel**)
T=14.86	58 -16 -4	left mid temporal cortex	4566
subcluster	sum classified voxels	subregion	
temporal	2096	superior t~ (r, 1491), Heschl (r, 214), mid-t~ (r, 200), rolandic operculum (r, 191)	
frontalparietal	758	insula (r, 758)	
**Cluster 3**	**x y z**	**region**	**size** (**voxel**)
T=12.23	10 34 -14	left gyrus rectus	713
subcluster	sum activated voxels	subregion	
frontal	272	medial orbitof~ (l, 147; r ,125)	
cingulate gyrus	110	anterior c~ (r, 110)	
**Cluster 4**	**x y z**	**region**	
T=10.15	−54 -70 20	left mid temporal cortex	377
subcluster	sum classified voxels	subregion	
occipital	228	mid o~ (l, 228)	
temporal	91	mid t~ (l, 91)	
**Cluster 5**			
T=7.43	46 -58 14	right mid temporal cortex	317
**Cluster 6**			
T=8.52	42 -56 -10	right inferior temporal cortex	226
**Cluster 7**			
T=6.05	36 -72 18	right mid temporal cortex	197
**Cluster 8**			
T=6.78	6 -58 -30	cerebellum	131
**Cluster 9**			
T=8.37	−34 -14 -22	right hippocampus	106
subcluster	sum classified voxels	subregion	
temporal	48	hippocampus (l, 48)	
fusiform/cuneus	21	fusiform (l, 21)	

*T*, statistic values; *l*, left; *r*, right; size, number of voxels, *c*~ cingulate, *f*~ frontal, *t*~ temporal, *p*~ parietal, xyz coordinates of the peak voxel (MNI space).

**Figure 5:**
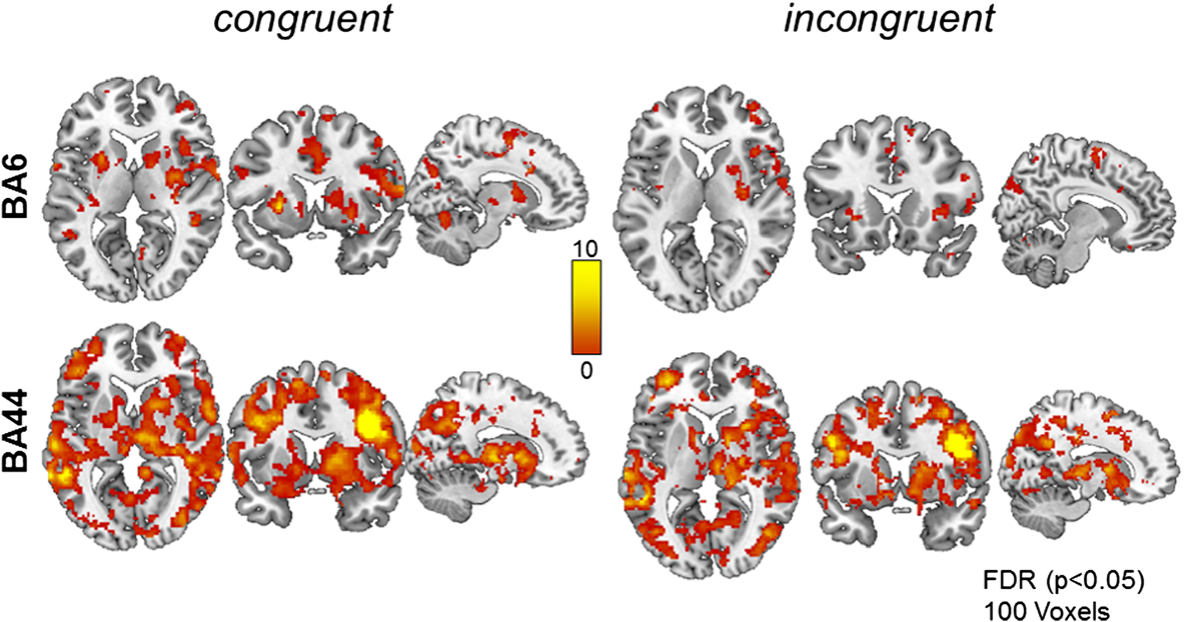
**fMRI connectivity results.** Additional connectivity analyses using the right Brodmann area 44 and the right Brodmann area 6 as seeds. As with the STS seed more widespread connectivity is observed for congruent stimuli, in particular for the BA 44 seed which included frontal and parietal cortical areas as well as basal ganglia and thalamus. This effect is less prominent for the BA 6 seed. FDR-corrected, p<0.05, minimum cluster size 100 voxels.

We also performed connectivity analyses using the right BA6 and the right BA44 as seed regions. The results are illustrated in Figure 5. The connectivity patterns obtained for these seed regions also revealed differences for congruent and incongruent stimuli. For the former, increased connectivity to basal ganglia and motor cortical areas was observed for congruent stimuli. This was more prominent for the Brodmann area 44 seed.

## Discussion

The present study asked two main questions: (a) To what extent congruent sonification accompanying movements improves perceptual processing of these movements, and (b) What are the brain systems supporting the processing of sonified movements?

The first question was addressed by the behavioural part of the study. Clearly, sonification led to a decisive advantage in the perceptual judgement task in that the errors associated with the comparison of the movement speed of the two video-segments of a trial were considerably smaller for congruent stimuli. Shams and Seitz [14] argued that, whereas “training on any pair of multisensory stimuli might induce a more effective representation of the unisensory stimulus, the effects could be substantially more pronounced for congruent stimuli.” They defined congruency as supported by “relationships between the senses found in nature. This spans the basic attributes such as concordance in space and time, in addition to higher-level features such as semantic content (e.g. object and speech information).” Indeed, in a perceptual learning experiment, in which one group was trained with congruent auditory–visual moving stimuli, the second group with incongruent auditory–visual stimuli and the third group with visual stimuli only, facilitation was specific to the congruent condition, thus ruling out a general alerting effect of the additional auditory stimulus [19]. The highly significant effect of congruency in the present study is a further proof for the benefit brought about by additional congruent sonification. It has to be kept in mind, however, that the present study used realistic biological motion stimuli with sonification based on kinematic parameters, whereas Kim et al. required the detection of coherently moving dots that were displaced and accompanied by a similar displacement of sound direction.

### fMRI

With regard to the neural underpinnings of the facilitatory effect of congruency fMRI showed marked differences between congruent and incongruent stimuli. The univariate analysis showed increased activation for congruent relative to incongruent stimuli in the superior and medial posterior temporal regions as well as the insula bilaterally and the precentral gyrus on the right side. The superior temporal region has been shown to be involved in multisensory processing in multiple studies. It receives converging auditory and visual inputs [20] and thus is equipped to contribute to multisensory integration [21-24]. Noesselt et al. [25] investigated trains of auditory and visual stimuli that either coincided in time or not. These authors found increased activation in STS when the visual stream coincided in time with the auditory stream and decreased activation for non-coincidence (using activation to unisensory stimuli as baseline). An influence of audiovisual synchrony has also been found in a number of other fMRI studies [26-29]. With regard to the audiovisual integration of speech stimuli for which the synchrony of lip-movements and sounds is of great importance again the caudal part of the superior temporal sulcus has been implicated [24,30,31]. A number of studies have revealed activation for audiovisual speech stimuli compared to their unimodal components presented separately [32,33]. It has further been shown that the visual component of audiovisual speech stimuli exerts a modulatory influence on the auditory areas located in the dorsal surface of the temporal lobe [34,35].

In light of these previous findings the increased activation in the superior temporal region for congruent stimuli in the univariate analysis suggests that audiovisual congruency leads to engagement of multisensory integration areas. This notion is further substantiated by the connectivity analysis (Figure 4B). Placing a seed in the left STS region revealed a widespread connectivity pattern for the congruent stimuli: Besides subcortical key players of the striato-thalamo-frontal motor-loops such as the caudate nucleus, putamen, thalamus and cerebellum, this network also included cortical regions in the medial superior frontal gyrus, superior, middle and inferior frontal gyrus, cingulate cortex, pre- and postcentral gyrus and parietal areas. By contrast, the incongruent stimuli engaged a much less widespread network. In particular, no connectivity was observed between the STS and the caudate nucleus and the putamen and the connectivity to the thalamus and cerebellum was less pronounced in comparison to the congruent stimuli. Also, with regard to cortical regions, incongruent stimuli showed a greatly reduced connectivity to frontal areas. This increased recruitment of basal ganglia and frontal motor-related areas was also seen for two additional seed areas (right Brodmann areas 6 and 44, Figure 5).

We would like to discuss the current patterns with regard to two topics: action observation and audiovisual integration. It has been proposed that the brain of an observer who observes someone else performing an action may simulate the performance [36] using a special neural system that has been termed the mirror neuron system [37-43]. The classical studies by Rizzolatti’s group have shown that the premotor and parietal cortex of monkeys harbours mirror neurons which discharge not only when the monkey performs an action but also when the monkey observes another monkey or an experimenter performing the same action [40,41,44]. Numerous brain imaging studies have suggested that a similar mirror neuron system exists in humans and comprises premotor cortex, parietal areas and the superior temporal sulcus (STS) [38,45-50]

With regard to the stimuli of the current study it is important that while observing the actions of an artificial handled to less activation of the mirror system than watching real hand actions [51,52], biomechanically possible actions (as used in the present study) give rise to robust activations compared to impossible movements [53]. Systematic manipulation of the stimuli further suggests that the human mirror system reflects the overlap between an observed action and the motor repertoire of the observer [54].

The current study revealed robust activation of major hubs of the human action observation system. In particular, the connectivity analysis showed that the STS during observation of the breast-stroking movement was intimately connected to frontal (including Brodmann areas 44 and 45) and parietal cortical areas that have been previously found in relation to action observation.

Importantly, we also found that congruent sonification compared to incongruent concurrent sounds led to increased activation in parts of the mirror neuron system including the frontal operculum, inferior parietal lobule and the superior temporal areas. The superior temporal area has been identified as being important for a number of complex cognitive processes: It has been found active during the processing of biological motion [55,56] and, emanating from this more basic capability, social perception [57-59]. As pointed out in the introduction, it has also been identified as important for audiovisual integration [25,60-62]. An integrative view of the functions of this area has been provided by Hein and Knight [63]. What is more, the connectivity analysis using the left STS as a seed region revealed a more robust and widespread connectivity for congruent compared to incongruent stimuli. Interestingly, trials with congruent sonification also showed connectivity to subcortical structures known to be part of the striato-thalamo-frontal motor loops, i.e. the caudate nucleus, putamen and the thalamus.

## Conclusion

This suggests that congruent sonification amplifies the neural activity of the action observation system. As shown in the behavioural part of this study, this enhanced neural representation of the observed movement leads to an improved perceptual analysis of the movement. Experiences in sports science also indicate that sonification of movements during exercise also results in improved, more precise performance of complex movements, such as rowing, golf driving, hammer throwing or swimming [12,64-69]. Further research needs to address whether athletes trained using movement sonification possess an enhanced representation of movements similar to professional musicians [4-7,70].

## Abbreviations

AE: Absolute error; BOLD: Blood oxygen level dependent; EPI: Echo planar imaging; fMRI: Functional magnetic resonance imaging; GLM: General linear model; STS: Superior temporal sulcus

## Competing interests

The authors declare that they have no competing interests.

## Authors’ contributions

GS, AE, TFM conceived the experimental design. BM, GS, AS performed the experiments. BM, AS, GS, AE, MH, TFM performed the analyses. AH and GS constructed the stimuli and the stimulation scenarios. GS, AE, TFM and BM wrote the various drafts of the manuscript. All authors read and approved the final manuscript.
